# The Expression Pattern of Adhesion G Protein-Coupled Receptor F5 Is Related to Cell Adhesion and Metastatic Pathways in Colorectal Cancer—Comprehensive Study Based on In Silico Analysis

**DOI:** 10.3390/cells11233876

**Published:** 2022-12-01

**Authors:** Huining Kang, Jakub Fichna, Ksenia Matlawska-Wasowska, Damian Jacenik

**Affiliations:** 1 Department of Internal Medicine, University of New Mexico Health Sciences Center, Albuquerque, NM 87131-0001, USA; 2 Department of Biochemistry, Faculty of Medicine, Medical University of Lodz, 92-215 Lodz, Poland; 3 Department of Pediatrics, University of New Mexico Health Sciences Center, Albuquerque, NM 87131-0001, USA; 4 Department of Pharmacology and Toxicology, University of Alabama at Birmingham, Birmingham, AL 35294-0019, USA; 5 Department of Cytobiochemistry, Faculty of Biology and Environmental Protection, University of Lodz, 90-236 Lodz, Poland

**Keywords:** adhesion G protein-coupled receptor F5, ADGRF5, G protein-coupled receptor 116, GPR116, colorectal cancer, metastasis, cell adhesion

## Abstract

Adhesion G protein-coupled receptor F5 (ADGRF5) is involved inthe neoplastic transformation of some cancer types. However, the significance of *ADGRF5* expression signature and the impact of signaling pathways mediated by ADGRF5 during neoplastic transformation of the colon and colorectal cancer (CRC) progression has been poorly examined. Using Gene Expression Omnibus and The Cancer Genome Atlas datasets, we showed that *ADGRF5* is overexpressed in the colons of patients with CRC. In line, combined analysis of *ADGRF5* expression with clinical characterization revealed an increased expression of *ADGRF5* in patients with more advanced stages of CRC compared to patients with early stages of CRC. The Spearman correlation analysis documented numerous genes positively and negatively correlated with the expression pattern of *ADGRF5* in the colon of patients with CRC. In the colon of CRC patients, the expression signature of *ADGRF5* was associated with genes participating in phosphatidylinositol 3-kinase/Akt, focal adhesion, cell adhesion molecules, and ribosome signaling pathways. Of note, *ADGRF5* expression correlated with the levels of tumor-infiltrating immune cells in the colon of CRC patients. Moreover, we found that CRC patients with high expression of *ADGRF5* had a significantly lower probability of overall survival and disease-free survival. In conclusion, our results support the prognostic value of *ADGRF5* and its potent therapeutic implication in CRC.

## 1. Introduction

Among several cancer types, colorectal cancer (CRC) is one of the most diagnosed cancer types in men and women worldwide. Most CRCs are sporadic, and an accumulation of genetic and epigenetic alterations is observed. Genomic alterations are responsible for oncogenes activation and/or tumor suppressor genes inactivation leading to the neoplastic transformation of the colon [1,2]. Several processes, such as chromosomal and microsatellite instability, as well as aberrant CpG methylation, are responsible for CRC development and progression. On the contrary, numerous signaling pathways such as mitogen-activated protein kinase, phosphatidylinositol 3-kinase (PI3K), transforming growth factor-β (TGF-β), WNT, and Notch pathways are deregulated in CRC and play crucial roles in the regulation of proliferation and migration as well as invasion of CRC cells [3,4,5,6,7]. In the past decades, several efforts have been made to improve the prevention of the neoplastic transformation of the colon and the treatment strategy for patients with CRC [2]. Despite many therapeutic approaches, such as surgical intervention, radiotherapy, and chemotherapy, including both classical and biological agents, many therapeutic strategies are not enough effective in the treatment of CRC patients. This clinical challenge seems to be directly associated with the late diagnosis of CRC. In fact, 86% of patients manifest some symptoms at the time of diagnosis that are directly related to more advanced disease stages [8]. Thus, additional efforts should be taken to improve CRC detection and treatment strategies for CRC patients.

Accumulating evidence highlights the role of adhesion G protein-coupled receptors (GPCRs) in multiple processes such as metabolism and immune response as well as several pathological conditions, including conditions that affect the cardiovascular, urinary, endocrine and nervous systems, respiratory and gastrointestinal tract systems, reproductive organs, and cancers, among others [9]. The adhesion GPCRs are a unique receptor subfamily in which amino acids sequences are composed of a GPCR proteolysis site and a highly conserved GPCR autoproteolysis-inducing domain which is responsible for the self-cleavage activity of adhesion GPCRs [9]. On the other hand, adhesion GPCRs are characterized by long extracellular fragments with multiple domains that mediate cell-cell and cell-matrix interaction [9]. Most adhesion GPCRs are orphan receptors; however, growing evidence suggests that adhesion GPCRs can act as typical GPCRs by stimulating numerous signaling pathways through modulation of G proteins activity [10].

The adhesion G protein-coupled receptor F5 (ADGRF5; also known as a G protein-coupled receptor 16, i.e., GPR116) is a member of family VI adhesion GPCRs, which contain immunoglobulin-like and immunoglobulin I-set domains as well as sperm protein, enterokinase, and agrin domain in the extracellular fragment [9]. ADGRF5 is essential in lung surfactant homeostasis. Specifically, it regulates surfactant lipid and protein accumulation in the alveolar space leading to labored breathing, which is directly associated with reduced lifespan [11,12,13]. Other studies indicate that ADGRF5 is engaged in the immune response, adipose function, glucose intolerance, and insulin resistance development, as well as angiogenesis [14,15,16]. Moreover, ADGRF5 affects the development and progression of breast and lung cancers as well as CRC [17,18,19,20,21,22,23]. Nevertheless, only limited efforts have been made to understand the roles of ADGRF5 in the neoplastic transformation of colon and CRC progression. Using Gene Expression Omnibus (GEO) and The Cancer Genome Atlas (TCGA) datasets, we studied *ADGRF5* expression patterns and the association between *ADGRF5* expression signature and immune cell infiltration as well as signaling pathways related to neoplastic transformation of colon and CRC progression.

## 2. Materials and Methods

### 2.1. Datasets and Patient Characteristics

The datasets from GEO (accession number: GSE21510, GSE32323, and GSE117606) were used to evaluate the expression of *ADGRF5* in the colon of controls and CRC patients. The level of *ADGRF5* expression in three GEO datasets was calculated using the z-score method independently in GSE21510, GSE32323, and GSE117606 datasets based on log2-transformed values. In total, 129 subjects (control, *n* = 25; CRC, *n* = 104) for GSE21510, 34 subjects (control, *n* = 17; CRC, *n* = 17) for GSE32323 and 139 subjects (control, *n* = 65; CRC, *n* = 74) for GSE117606 were enrolled in the study. The dataset and corresponding clinical characteristics of CRC patients were obtained from TCGA, Firehose Legacy from cBioPortal for Cancer Genomic [24,25]. In total, 382 subjects were employed in the study. Microarray gene expression data described as an RNA Seq v2 mRNA median z-score were filtered for missing values and used in the analysis of *ADGRF5* expression pattern and survival analysis of patients with CRC.

### 2.2. Correlation Analysis

Positive and negative correlations between the expression of *ADGRF5* and the other genes were estimated using the co-expression tool in cBioPortal for the Cancer Genomic and TCGA, Firehose Legacy dataset. Genes correlated positively with *ADGRF5* expression and characterized by Spearman value higher or equal to 0.50 were employed in further analysis. The top 1000 genes negatively correlated with the expression of *ADGRF5* were used in subsequent analysis. To note, genes negatively correlated with the expression of *ADGRF5* were characterized by an *R*-value of the correlation co-efficiency significantly lower than 0.50, and for this reason, the top 1000 genes negatively correlated with the expression of *ADGRF5* in the colon of CRC patients was obtained.

### 2.3. Pathway Enrichment Analysis

Pathway enrichment analyses were performed using selected genes correlated with the expression of *ADGRF5,* which were identified in the TCGA, Firehose Legacy dataset. Positively and negatively correlated genes with *ADGRF5* expression patterns were classified into signaling pathways using Database for Annotation, Visualization, and Integrated Discovery (DAVID) functional annotation analysis [26,27]. Gene lists included positively and negatively correlated genes with *ADGRF5* expression and were uploaded separately using the official gene symbol with limited species to *Homo sapiens* in the background. The Kyoto Encyclopedia of Genes and Genomes (KEGG) pathway was used to evaluate signaling pathways based on the most correlated genes with the expression of *ADGRF5* documented in TCGA, Firehose Legacy dataset.

### 2.4. Gene Expression Profiling

Gene expression data for 55 genes that positively correlated and 86 genes negatively correlated with the expression of *ADGRF5* were ranked according to *ADGRF5* expression signature in the colon of CRC patients derived from TCGA, Firehose Legacy dataset. Hierarchical clustering was performed in Cluster 3.0 using average linkage. Java TreeView 3.0 were used for the visualization of genes correlated with the expression of *ADGRF5* belonging to the most significant signaling pathways.

### 2.5. Survival Analysis

The association of *ADGRF5* expression signature with the probability of overall survival (OS) and disease-free survival (DFS) was assessed by examining the difference in survival between patients with high and low *ADGRF5* expressions. The high expressions are above the upper tertile, and the low expressions are below the lower tertile. The Kaplan-Meier method was used to estimate OS and DFS probability, and the Log-rank test was used to compare the OS or DFS probabilities between the patient groups.

### 2.6. Immune Cell Infiltration Analysis

TIMER 2.0 was used to study the correlation between the expression of *ADGRF5* and immune cell infiltration in patients with CRC [28,29,30]. Systematic analysis of tumor-infiltrating immune cells was limited to the TIMER algorithm and immune cell types with a *p*-value < 0.05.

### 2.7. Statistical Analysis

Descriptive statistics were used to summarize the associations of *ADGRF5* expression with the clinical characteristics of patients with CRC. Median ± interquartile range and mean ± standard deviation (SD) of *ADGRF5* expression were used to estimate the *ADGRF5* expression in the GEO and TCGA datasets, respectively. A Non-parametric Mann-Whitney U test was used to detect the difference of each variable. The Spearman correlation coefficient was used to identify genes whose expression was associated with that of the *ADGRF5* expression signature. All the analyses were performed using GraphPad Prism 5.0 and R software.

## 3. Results

### 3.1. ADGRF5 Is Overexpressed in the Colon of Patients with Colorectal Cancer

**Figure 1:**
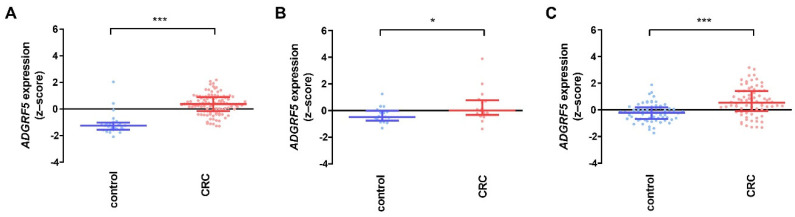
*ADGRF5* expression pattern in the colon of controls and CRC patients derived from GSE21510 ((**A**); control, *n* = 25; CRC, *n* = 104), GSE32323 ((**B**); control, *n* = 17; CRC, *n* = 17), and GSE117606 ((**C**); control, *n* = 65; CRC, *n* = 74) datasets. Data are presented as medians with interquartile range; * *p* < 0.05, *** *p* < 0.001.

The *ADGRF5* expression pattern was estimated using GSE21510, GSE32323, and GSE117606 datasets obtained from GEO, and the expression of *ADGRF5* was evaluated in each dataset independently. As shown in Figure 1A–C, a statistically significant higher copy number of the *ADGRF5* gene in the colon of CRC patients compared to controls (*p* < 0.05 for GSE32323; *p* < 0.001 for GSE21510 and GSE117606) was documented. 

### 3.2. ADGRF5 Expression Is Related to Clinical Characterization of Patients with Colorectal Cancer

In order to further explore the significance of *ADGRF5* expression signature in the colon of CRC patients, we assembled a large collection of CRC patients using the Firehose Legacy dataset available at TCGA. The data from the dataset for *ADGRF5* expression and corresponding clinical characterization of CRC patients were used. The results are shown in Table 1. The expression of *ADGRF5* tended to be higher in the colon of black or African American patients with CRC (*p* = 0.058) compared to white patients with CRC. We found an increased expression of *ADGRF5* in CRC patients with a mucinous type of colon adenocarcinoma compared to colon adenocarcinoma (*p* < 0.01). *ADGRF5* expression changes were documented only in CRC patients with tumors localized in the colon but not in the rectum. Moreover, in the colon of patients with CRC with more advanced stages, classified as III and IV stage, the expression of *ADGRF5* was higher than in patients with CRC stages corresponding to I and II stage (*p* < 0.05). Analysis of *ADGRF5* expression in CRC patients according to tumor-node-metastasis (TNM) staging system revealed higher expression of *ADGRF5* when the extent of the tumors was classified as a T3 and T4 compared to the tumors described as a T1 and T2 (*p* < 0.01). Significantly higher copy numbers of the *ADGRF5* gene (*p* < 0.05) were found in CRC patients with nearby lymph nodes affected by tumor cells (N1/2) in relation to CRC patients, which were characterized by a lack of tumor cells in the nearby lymph nodes (N0). Moreover, we did not identify statistically significant differences in the expression of *ADGRF5* according to gender, age, race, or histological diagnosis for rectum in patients with CRC. Collectively, these results indicate that *ADGRF5* participates in the progression of CRC.

### 3.3. ADGRF5 Expression Is Positively Correlated with Genes Participating in Cell Adhesion and Metastatic Pathways in Colorectal Cancer

We next performed correlation analysis using the above-mentioned dataset provided by TCGA, and we identified numerous genes positively correlated with the expression of *ADGRF5* in the colon of CRC patients. We found 500 genes positively correlated with the expression of *ADGRF5* and characterized by Spearman R-value higher or equal to 0.50 in the TCGA, Firehose Legacy dataset.

**Figure 2:**
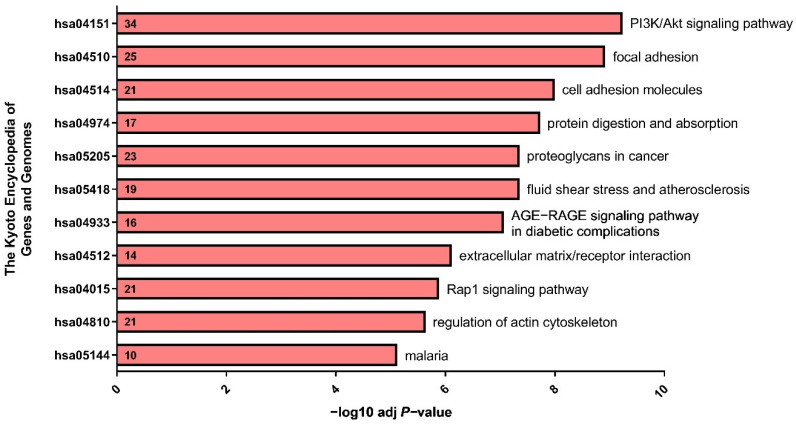
Scheme representing the number of genes positively correlated with *ADGRF5* expression pattern in the colon of CRC patients and genes correlated with *ADGRF5* expression pattern involved in the signaling pathways.

To further explore the functional significance of the 500 genes identified, we performed signaling pathway analyses using DAVID functional annotation and KEGG pathway analysis tools. The genes identified were significantly enriched in PI3K/Akt (hsa04151), focal adhesion (hsa04510), and cell adhesion molecules (hsa04514) signaling pathways (Figure 2). The symbol, ID, and description of genes representing PI3K/Akt, focal adhesion and cell adhesion molecules signaling pathways, and *R*-value of the correlation co-efficiency are presented in Table 2, Table 3 and Table 4, respectively.

In the PI3K/Akt signaling pathway we found genes such as serine/threonine kinase 3, i.e., *AKT3*, angiopoietin (*ANGPT*) 1 and 2, collagen type I α2 chain (*COL1A1*), collagen type III α1 chain (*COL3A1*), collagen type IV type α1 (*COL4A1*) and collagen type IV α2 chain (*COL4A2*), collagen type V α1 chain (*COL5A1*), collagen type V α2 chain (*COL5A2*) and collagen type V α3 chain (*COL5A3*), collagen type VI α3 chain (*COL6A3*), collagen type XI α1 chain (*COL11A1*), collagen type XXIV α1 chain (*COL24A1*), coagulation factor II thrombin receptor (*F2R*), fibroblast growth factor (*FGF*) 1 and 7, FMS-related receptor tyrosine kinase 1 (*FLT1*), G protein subunit β4 (*GNB4*), G protein subunit γ (*GNG*) 2 and 11, hepatocyte growth factor (*HGF*), integrin subunit α (ITGA) 1, 4, 5, and V, integrin subunit β (*ITGB*) 1 and 3, kinase insert domain receptor (*KDR*), laminin subunit α4 (*LAMA4*), laminin subunit γ1 (*LAMC1*), oncostatin M receptor (*OSMR*), platelet-derived growth factor C (*PDGFC*), platelet-derived growth factor receptor α (*PDGFRA*), and β (*PDGFRB*), serum/glucocorticoid regulated kinase 1 (*SGK1*), TEK receptor tyrosine kinase (*TEK*), thrombospondin 2 (*THBS2*), tenascin C (*TNC*), Toll-like receptor 2 (*TLR2*), and vascular endothelial growth factor C (*VEGFC*).

In the colons of CRC patients, we found genes such as *AKT3*, caveolin (*CAV*) 1 and 2, *COL1A2*, *COL3A1*, *COL4A1*, *COL4A2*, *COL5A1*, *COL5A2*, *COL5A3*, *COL6A3*, *COL11A1*, *COL24A1*, *FLT1*, *HGF*, *ITGA1*, *ITGA4*, *ITGA5*, *ITGAV*, *ITGB1*, *ITGB3*, *KDR*, *LAMA4*, *LAMC1*, myosin light chain kinase (*MYLK*), *PDGFC*, *PDGFRA*, *PDGFRB*, *THBS2*, *TNC* and *VEGFC* which are characterized by a positive correlation with *ADGRF5* expression pattern, and participate in focal adhesion signaling pathway (Table 3).

In the cell adhesion molecules signaling pathway, we identified numerous genes positively correlated with ADGRF5 expression signature such as CD34 (*CD34*) and CD86 (*CD86*) molecules, cadherin (*CDH*) 2 and 5, endothelial cell adhesion molecule (*ESAM*), *ITGA4*, *ITGAV*, *ITGB1*, junctional adhesion molecule (*JAM*) 2 and 3, neural cell adhesion molecule 2 (*NCAM2*), neuroligin 4 X-linked (*NLGN4X*), programmed cell death 1 ligand 2 (*PDCD1LG2*), platelet and endothelial cell adhesion molecule 1 (*PECAM1*), protein tyrosine phosphatase receptor (*PTPR*) type C and M, syndecan 2 (*SDC2*), selectin (*SEL*) E and L, vascular cell adhesion molecule 1 (*VCAM1*) and versican (*VCAN*) (Table 4).

**Figure 3:**
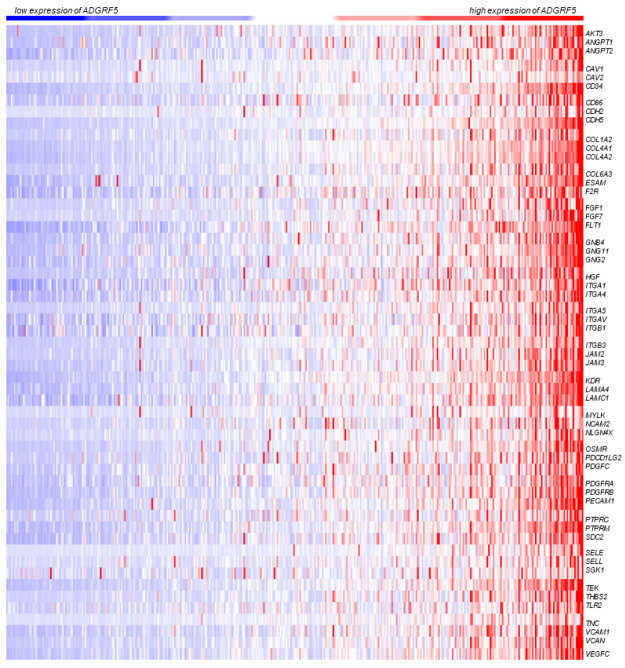
The heatmaps representing the expression of ADGRF5 and genes belonging to PI3K/Akt (hsa04151), focal adhesion (hsa04510), and cell adhesion molecules (hsa04514) signaling pathways determined in the colon of CRC patients derived from TCGA, Firehose Legacy dataset.

Subsequently, we next performed expression profiling of 55 genes participating in PI3K/Akt, focal adhesion, and cell adhesion molecules signaling pathways according to *ADGRF5* expression ranked from low to high expression of *ADGRF5*. As shown in Figure 3, the expression pattern of genes representing the above-mentioned signaling pathways was similar to the expression profile of *ADGRF5* in the colon of CRC patients (Figure 3).

### 3.4. ADGRF5 Expression Is Negatively Correlated with Genes Participate in Ribosome Pathway in Colorectal Cancer

DAVID functional annotation analysis and KEGG pathway analysis revealed that the top 1000 genes negatively correlated with the expression of *ADGRF5* are involved in various signaling pathways, including ribosome (hsa03010), which was the most significant signaling pathway (adjusted *p* < 0.00001) associated with the expression signature of *ADGRF5* in CRC patients. The symbol and description of genes enriched in ribosome signaling pathways and the *R*-value of the correlation co-efficiency are presented in Table 5. In the ribosome signaling pathway, we identified negatively correlated genes such as FAU ubiquitin-like and ribosomal protein S30 fusion (*FAU*), mitochondrial ribosomal protein L (*MRPL*) 1, 2, 3, 4, 11, 12, 14, 15, 16, 17, 20, 21, 22, 23, 24, 27, 32, 34 and 36, mitochondrial ribosomal protein S (*MRPS*) 2, 5, 9, 12, 15, 16, 17 and 18A, ribosomal protein L (*RPL*) 3, 5, 6, 7A, 8, 11, 12, 13, 14, 15, 18, 18A, 19, 23, 23A, 24, 27, 27A, 28, 29, 31, 32, 34, 35, 35A, 36, 37, 37A, 38 and 39, ribosomal protein lateral stalk subunit *p* (*RPLP*) 0, 1 and 2, ribosomal protein S (*RPS*) 2, 3, 4X, 6, 7, 8, 9, 10, 11, 13, 14, 15, 15A, 16, 17, 18, 19, 21, 23, 24, 25, 27A, 29 and A, as well as ubiquitin A-52 residue ribosomal protein fusion product 1 (*UBA52*).

**Figure 4:**
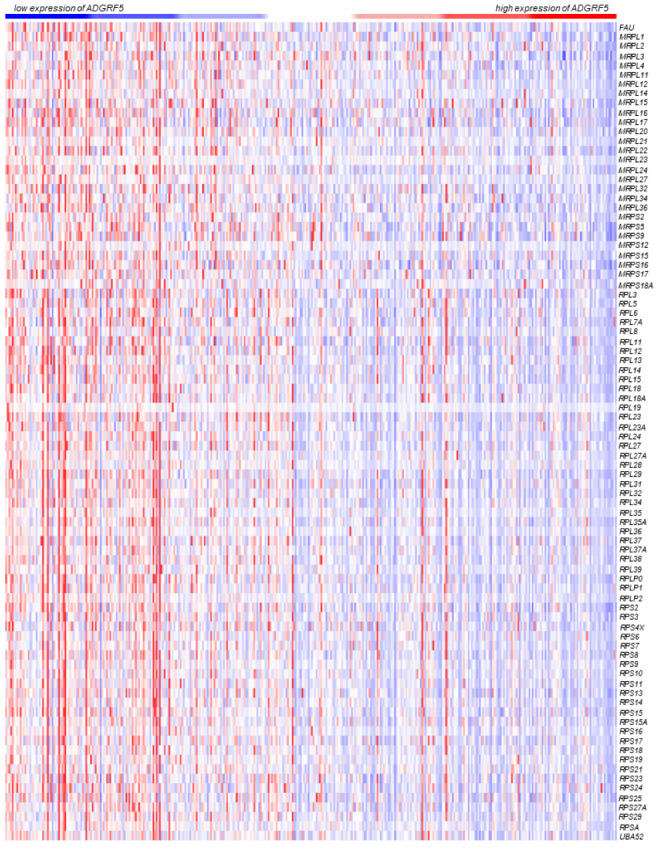
The heatmaps representing the expression of *ADGRF5* and genes belonging to the ribosome (hsa03010) signaling pathways determined in the colon of CRC patients derived from the TCGA, Firehose Legacy dataset.

Next, we ranked the expression of 86 genes in the ribosome signaling pathway according to the expression pattern of *ADGRF5* in the colon of patients with CRC. As shown in Figure 4, lower expression of *ADGRF5* was associated with higher expression of genes participating in the ribosome signaling pathway, and conversely, higher expression of *ADGRF5* was associated with lower expression of genes regulating ribosome. Nevertheless, with weak correlation co-efficiency for genes related to ribosome signaling pathway, the expression pattern of genes negatively correlated with the expression of *ADGRF5* is not so pronounced as in the case of genes positively correlated with *ADGRF5* expression signature.

### 3.5. Increased Expression Pattern of ADGRF5 in the Colon of Patients with CRC Is Associated with Poor Probability of Overall Survival and Disease-Free Survival

**Figure 5:**
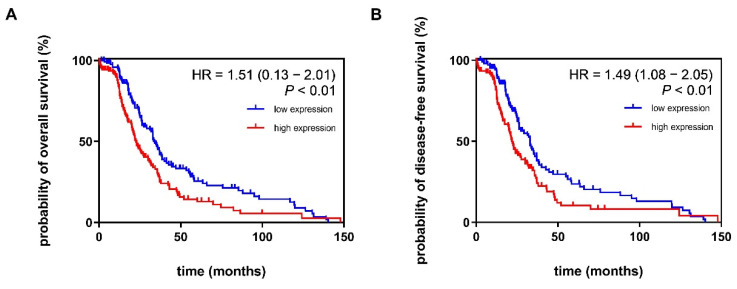
(**A**) Probability of overall survival and (**B**) disease-free survival of patients with low (blue) and high (red) expression of *ADGRF5* in the colon of patients with CRC.

We investigated the association of *ADGRF5* expression signature with CRC patient survival using data obtained from the TCGA, Firehose Legacy dataset. We found that the expression of *ADGRF5* in the colon of CRC patients was associated with the probability of both OS and DFS. As shown in Figure 5A,B, higher expression of *ADGRF5* was accompanied by a worse probability of OS and DFS in CRC patients, which was confirmed by HR = 1.51 (*p* < 0.01) and HR = 1.49 (*p* < 0.01) for CRC patients, respectively. To note, CRC patients with higher expression of *ADGRF5* were characterized by a median survival time of 34 months for OS and 32 months for DFS when compared to CRC patients with lower expression of *ADGRF5* whose median survival time was evaluated as 22 months for both OS and DFS. 

### 3.6. Expression Pattern of ADGRF5 Is Correlated with the Levels of Tumor-Infiltrating Immune Cells in the Colon of Patients with CRC

**Figure 6:**
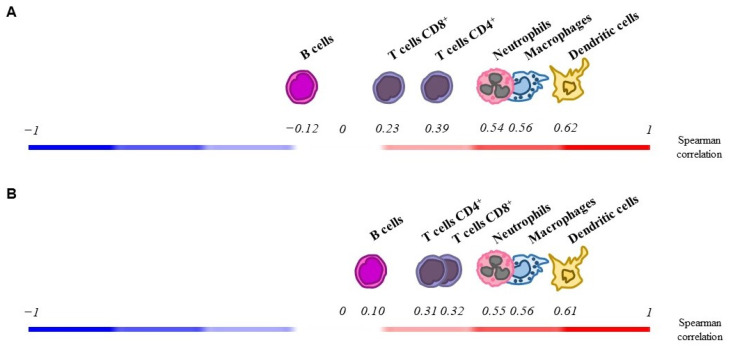
Correlation between *ADGRF5* expression and the level of immune cell infiltration in colon (**A**) and rectal adenocarcinoma (**B**).

Finally, we evaluated the association between *ADGRF5* expression and the level of immune cell infiltration in the colon of CRC patients using TIMER 2.0. Among distinct types of immune cells, the levels of dendritic cells (*R* = 0.62), macrophages (*R* = 0.56), neutrophils (*R* = 0.54), and CD4^+^ (*R* = 0.39), as well as CD8^+^ (*R* = 0.23) T cells infiltration were positively correlated (*p* < 0.05) with the expression of *ADGRF5* in the colon of patients with adenocarcinoma (Figure 6A). On the other hand, the level of B cells infiltration was negatively correlated (*R* = −0.12, *p* < 0.05) with the expression of *ADGRF5* in the colon of patients with adenocarcinoma (Figure 6A). As shown in Figure 6B, the levels of dendritic cells (*R* = 0.61), macrophages (*R* = 0.56), neutrophils (*R* = 0.55), CD8^+^ (*R* = 0.32), and CD4^+^ (*R* = 0.31) T cells, as well as B cells (*R* = 0.10) infiltration, were positively correlated (*p* < 0.05) with the expression of *ADGRF5* in the rectum of patients with adenocarcinoma.

## 4. Discussion

According to The Human Protein Atlas, *ADGRF5* is widely expressed in many types of human tissues, which suggests that signaling pathways mediated by *ADGRF5* may be involved in the regulation of both physiological and pathophysiological conditions. Accumulating evidence indicates that *ADGRF5* is a crucial member of adhesion GPCRs that mediate numerous processes such as proliferation, apoptosis, and migration, as well as invasion in the progression of some cancer types [17,18,19,20,21,22,23]. In fact, Tang et al. identified that ADGRF5 acts as a critical regulator of breast cancer metastasis. An increased *ADGRF5* expression is associated with breast cancer progression and recurrence and poor prognosis of patients with breast cancer [18]. It should also be noted that the significance of *ADGRF5* in breast cancer was evaluated *in vitro* and *in vivo*, and both approaches confirmed that *ADGRF5* depletion in breast cancer cells was related to the reduced ability of breast cancer cells to migrate and invade [18].

Here, by employing three independent GEO datasets, we demonstrated that *ADGRF5* is overexpressed in the colon of patients with CRC when compared to controls. In addition, the analysis of the *ADGRF5* expression signature in the TCGA, Firehose Legacy dataset revealed a stage-dependent increase of *ADGRF5* expression in CRC patients, and *ADGRF5* expression patterns were associated with the progression of CRC. Yang et al. linked the deregulation of *ADGRF5* expression to the differentiation stage and distant metastasis in CRC [19]. In our study, we demonstrated that the *ADGRF5* expression pattern is associated not only with the CRC stage but also with lymph node metastasis, suggesting that *ADGRF5* may be involved in the regulation of epithelial-mesenchymal transition of CRC cells. In addition, we found an increased expression of *ADGRF5* in patients with a mucinous type of colon adenocarcinoma compared to colon adenocarcinoma. Colon mucinous adenocarcinoma is manifested by the presence of abundant mucous secretion comprising at least 50% of the tumor volume [31]. Mucin 2 and 5AC are two major types of mucins that are involved in the development of colon mucinous adenocarcinoma. However, the mechanisms by which both mucins regulate CRC progression are poorly understood. Clinically, colon mucinous adenocarcinoma is associated with worse clinical characteristics and worse prognosis compared to patients with colon adenocarcinoma [32,33]. Collectively, our findings suggest that CRC progression co-exists with *ADGRF5* overexpression in the colon of CRC patients, suggesting the roles of *ADGRF5* in the development and progression of CRC.

Little is known about the possible mechanisms responsible for the induction of *ADGRF5* expression in cancer. Among several post-transcriptional mechanisms, RNA interference seems to be involved in the regulation of *ADGRF5* gene expression in CRC. Studies by Wang et al. demonstrated that *ADGRF5* is a direct target for miR-511-5p [21]. Functionally, miR-511-5p mediates neoplastic transformation and progression of lung squamous cell carcinoma and CRC, as well as playing a role in the regulation of immune response by monocytes [21,34,35]. In CRC, miR-511-5p acts as a tumor suppressor microRNA, and the expression of miR-511-5p is reduced in both CRC tissues and cell lines compared to the controls. Moreover, miR-511-5p expression is inversely correlated with the differentiation of CRC and TNM stages of CRC patients. Furthermore, survival analysis revealed that CRC patients with low expression of miR-511-5p are characterized by a poor probability of OS and DFS [21]. It was estimated that the 3′UTR region of the *ADGRF5* mRNA sequence contains the binding site for miR-511-5p and *ADGRF5*-miR-511-5p interaction seems to be responsible for modulation of proliferation, apoptosis, and invasion of CRC cells. *In vitro* studies on HCT116 and LOVO cells conducted by Yang et al. also noted that silencing of the *ADGRF5* gene was accompanied by the reduced ability of CRC cells to proliferate and invade [19]. Above mentioned processes seem to be mediated by the regulation of both Akt and extracellular signal-regulated kinase (ERK) activity. In fact, *in vitro* analysis using CRC cells and siRNA against *ADGRF5* documented that the silencing of *ADGRF5* led to a reduction of CDH2, Snail family zinc finger transcriptional factor, phospho-Akt, and phospho-ERK 1/2 protein level suggesting that its role in the progression of CRC [19].

The main hallmark of cancer progression is epithelial-mesenchymal transmission underlying cancer cells’ migratory and invasive capabilities. Accumulating evidence indicates that numerous signaling pathways participate in epithelial-mesenchymal transmissions, such as TGF-β, receptor tyrosine kinase, extracellular matrix, or PI3K/Akt, among others [36]. Our study documented a strong positive correlation between *ADGRF5* expression pattern and expression of a number of genes involved in PI3K/Akt, focal adhesion, and cell adhesion molecules signaling pathways. Then PI3K/Akt signaling pathway is a major effector of epithelial-mesenchymal transmission and immunosuppression, and according to numerous reports, the action of the PI3K/Akt signaling pathway in cancers is multidirectional. In CRC, the hyperactivated PI3K/Akt signaling pathway positively regulates transcription mediated by NF-κB and β-catenin, which in turn are responsible for the pro-tumorigenic and invasive capacity of cancer cells [4,37,38]. It has to be highlighted that *ADGRF5* may mediate the pro-tumorigenic action of the PI3K/Akt pathway through G protein activity regulation. The previous findings by Tang et al. documented that *ADGRF5* affects signaling pathways responsible for breast cancer progression coupling to Gαq [18]. On the contrary, the PI3K/Akt signaling pathway plays a role in the negative regulation of the *CDH1* gene encoding E-cadherin, which is a crucial regulator of cell adhesion and tight junctions and serves as a marker of epithelial phenotype cells [39,40]. In fact, mesenchymal-type cancer cells have low expression of epithelial markers like *CDH1* and high expression of mesenchymal markers such as *CDH2* gene encoding N-cadherin, fibronectin, and vimentin, among others. Additionally, invasive cancer cells are characterized by a lack of polarization and decreased cell adhesion, as well as disturbed cell-cell and cell-matrix interactions [39]. Cadherins, caveolins, collagens, integrins, junctional adhesion molecules, laminins, and selectins all maintain proper cell function and physiological morphology of cells. Observational studies documented aberrant expression of *CAV1*, *COL1A2*, *COL3A1*, *COL5A2*, *COL6A3*, *COL11A1*, *ITGA1*, *ITGA5*, *ITGB1*, *LAMA4*, *JAM2*, *JAM3*, and *VCAM1* in CRC, among others [41,42,43,44,45,46,47,48,49,50,51,52,53]. Numerous of the above-mentioned genes represent focal adhesion regulators and extracellular matrix/receptor interaction signaling molecules. The expression of these genes was positively correlated with the expression of *ADGRF5* signature in the colon of patients with CRC in our studies (refer to Table 2, Table 3 and Table 4). Our results suggest that *ADGRF5* may participate in cell junction machinery and may be involved in epithelial-mesenchymal transmission during the development of CRC. Nevertheless, further studies employing *in vitro* and *in vivo* approaches are required to dissect the mechanism by which *ADGRF5* contributes to the change of epithelial to mesenchymal phenotype of cells during CRC progression.

In the colon of patients with CRC, we identified a negative correlation between *ADGRF5* expression and the expression of 86 genes of the ribosome signaling pathway. It should also be noted that the *R*-value describing the Spearman correlation between ribosomal genes was lower than for genes positively correlated with *ADGRF5* expression signature, and weak to moderate association between *ADGRF5* expression pattern and ribosomal genes expression was documented. Nevertheless, in CRC, alteration of various ribosomal genes is recognized, which indicates that ribosomal stress contributes to CRC development and progression. Indeed, ribosomal protein genes contribute to processes such as proliferation, apoptosis, hypoxia, glycolysis, and cell cycle regulation during neoplastic transformation of the colon, and their action seems to be associated, for instance, with the formation of ribosomal subunits or rRNA processing [54,55,56,57].

Finally, using Kaplan-Meier survival curves, we analyzed the clinical significance of *ADGRF5* expression signature in patients with CRC and showed that CRC patients with increased expression of *ADGRF5* in the colon had an inferior probability of OS and DFS compared to CRC patients with reduced *ADGRF5* expression in the colon. The results from survival and pathway enrichment analyses indicate that *ADGRF5* may modulate CRC progression in patients promoting pro-metastatic pathways. Additionally, our analyses document the link between *ADGRF5* expression and the levels of tumor-infiltrating immune cells in the colon and rectum of patients with adenocarcinomas. The levels of immune cell infiltration and composition of immune cells in the tumor microenvironment are crucial for patients’ survival and cancer prognosis. However, conflicting results regarding the tumor-infiltrating cells seem directly related to tumor microenvironment plasticity, which is continuously changing in response to signals from surrounding tissues. Nevertheless, accumulating evidence highlighted the crucial role of T cells, neutrophils, and macrophages, as well as dendritic cells, in the development and progression of CRC. Wang et al. found that the levels of T cells may predict the treatment efficiency and OS as well as progression-free survival in CRC patients [58]. On the contrary, fibroblasts, macrophages, and neutrophils may support tumor growth and the invasion ability of cancer cells. For instance, neutrophils are one of the major sources of matrix metallopeptidase 9, which promotes extracellular matrix remodeling and angiogenesis [59,60]. Furthermore, macrophages also affect the above-mentioned processes, as evidenced by their role in the regulation of proteins related to epithelial-mesenchymal transition [61]. In our study, dendritic cells showed the strongest correlation with the expression of *ADGRF5* among all the tested types of immune cells. Strikingly, studies by Sandel et al. showed that tumor-infiltrating dendritic cells affected local tumor cell and immune cell interactions, and their levels were associated with prognosis in CRC patients [62]. The link between *ADGRF5* and inflammation was pointed out in the study performed by Kubo et al. [63]. Our evidence from immune cell infiltration analysis in the colon of CRC patients supports previous findings about *ADGRF5* function in immunity. Moreover, correlation analysis indicated that *ADGRF5* expression is associated with genes related to immune response, such as *TLR2,* which plays a fundamental role in the activation of innate immune response, among others [64].

Nevertheless, the role of ADGRF5 in both immunomodulation and neoplastic transformation of the colon remains elusive. Thus, the results of our comprehensive *in silico* study provide a basis for further investigation of the roles of *ADGRF5* in CRC and other tumors.

## 5. Conclusions

In this study, we identified that *ADGRF5* is overexpressed in the colons of patients with CRC, and *ADGRF5* expression patterns are associated with the disease stage. Moreover, patients with high expression of *ADGRF5* are characterized by a shorter probability of overall and disease-free survival. Our results suggest that ADGRF5 may play a role in epithelial-mesenchymal transmission and may be responsible for the modulation of cell adhesion and metastatic pathways during the neoplastic transformation of the colon, and CRC progression. Further studies on identifying *ADGRF5*-mediated pathways are warranted to develop a better understanding of the roles of *ADGRF5* in the development and progression of CRC.

## Figures and Tables

**Table 1:** *ADGRF5* expression pattern in the colon of colorectal cancer patients in relation to clinical characteristics.

Characteristic of Patients with Colorectal Cancer	*ADGRF5* Expression	*p*-Value	Number of Subjects
Sex			
Men	−0.0406 ± 0.779	ns	207
Women	0.0470 ± 1.226		169
Age			
Below the age of 50	0.1844 ± 1.140	ns	57
Above the age of 50	−0.0343 ± 0.976		319
Race			
Black or African American	0.1247 ± 1.804	ns	60
White	−0.0109 ± 0.770		270
Site of tumor			
Colon	−0.0428 ± 1.033	*	283
Rectum	0.1219 ± 0.913		91
Histological diagnosis—colon			
Colon adenocarcinoma	−0.1569 ± 0.612	**	242
Colon mucinous adenocarcinoma	0.7217 ± 2.233		38
Histological diagnosis—rectum			
Rectal adenocarcinoma	0.0855 ± 0.736	ns	86
Rectal mucinous adenocarcinoma	0.748 ± 2.596		5
Stage			
I/II	−0.0861 ± 1.041	*	191
III/IV	0.0762 ± 0.905		166
TNM—tumor			
1/2	−0.2936 ± 0.525	**	67
3/4	0.0644 ± 1.074		307
TNM—node			
0	−0.0999 ± 1.011	*	205
1/2	0.1131 ± 0.994		168
TNM—metastasis			
0	−0.0513 ± 0.788	ns	256
1	0.1005 ± 0.753		50

Values of *ADGRF5* expression are presented as a means of z-scores ± SD; ns—not significant, * *p* < 0.05, ** *p* < 0.01.

**Table 2:** Genes positively correlated with *ADGRF5* expression in the colon of colorectal cancer patients belonging to the PI3K/Akt signaling pathway—hsa04151.

Gene Symbol	Gene ID	Gene Description	Spearman Correlation
*AKT3*	10000	Serine/threonine kinase 3	0.80
*ANGPT1*	284	Angiopoietin 1	0.66
*ANGPT2*	285	Angiopoietin 2	0.74
*COL1A2*	1278	Collagen type I α2 chain	0.71
*COL4A1*	1282	Collagen type IV α1 chain	0.92
*COL4A2*	1284	Collagen type IV α2 chain	0.89
*COL6A3*	1293	Collagen type VI α3 chain	0.78
*F2R*	2149	Coagulation factor II thrombin receptor	0.72
*FGF1*	2246	Fibroblast growth factor 1	0.70
*FGF7*	2252	Fibroblast growth factor 7	0.71
*FLT1*	2321	FMS-related receptor tyrosine kinase 1	0.81
*GNB4*	59345	G protein subunit β4	0.78
*GNG2*	54331	G protein subunit γ2	0.75
*GNG11*	2791	G protein subunit γ11	0.76
*HGF*	3082	Hepatocyte growth factor	0.72
*ITGA1*	3672	Integrin subunit α1	0.71
*ITGA4*	3676	Integrin subunit α4	0.74
*ITGA5*	3678	Integrin subunit α5	0.79
*ITGAV*	3685	Integrin subunit αV	0.63
*ITGB1*	3688	Integrin subunit β1	0.61
*ITGB3*	3690	Integrin subunit β3	0.83
*KDR*	3791	Kinase insert domain receptor	0.88
*LAMA4*	3910	Laminin subunit α4	0.83
*LAMC1*	3915	Laminin subunit γ1	0.74
*OSMR*	9180	Oncostatin M receptor	0.81
*PDGFC*	56034	Platelet-derived growth factor C	0.65
*PDGFRA*	5156	Platelet-derived growth factor receptor α	0.69
*PDGFRB*	5159	Platelet-derived growth factor receptor β	0.80
*SGK1*	6446	Serum/glucocorticoid regulated kinase 1	0.50
*TEK*	7010	TEK receptor tyrosine kinase	0.85
*THBS2*	7058	Thrombospondin 2	0.68
*TLR2*	7097	Toll-like receptor 2	0.59
*TNC*	3371	Tenascin C	0.71
*VEGFC*	7424	Vascular endothelial growth factor C	0.78

**Table 3:** Genes positively correlated with *ADGRF5* expression in the colon of colorectal cancer patients belonging to focal adhesion signaling pathway—hsa04510.

Gene Symbol	Gene ID	Gene Description	Spearman Correlation
*AKT3*	10000	Serine/threonine kinase 3	0.80
*CAV1*	857	Caveolin 1	0.78
*CAV2*	858	Caveolin 2	0.58
*COL1A2*	1278	Collagen type I α2 chain	0.71
*COL4A1*	1282	Collagen type IV α1 chain	0.92
*COL4A2*	1284	Collagen type IV α2 chain	0.89
*COL6A3*	1293	Collagen type VI α3 chain	0.78
*FLT1*	2321	FMS-related receptor tyrosine kinase 1	0.81
*HGF*	3082	Hepatocyte growth factor	0.72
*ITGA1*	3672	Integrin subunit α1	0.71
*ITGA4*	3676	Integrin subunit α4	0.74
*ITGA5*	3678	Integrin subunit α5	0.79
*ITGAV*	3685	Integrin subunit αV	0.63
*ITGB1*	3688	Integrin subunit β1	0.61
*ITGB3*	3690	Integrin subunit β3	0.83
*KDR*	3791	Kinase insert domain receptor	0.88
*LAMA4*	3910	Laminin subunit α4	0.83
*LAMC1*	3915	Laminin subunit γ1	0.74
*MYLK*	4638	Myosin light chain kinase	0.68
*PDGFC*	56034	Platelet-derived growth factor C	0.65
*PDGFRA*	5156	Platelet-derived growth factor receptor α	0.69
*PDGFRB*	5159	Platelet-derived growth factor receptor β	0.80
*THBS2*	7058	Thrombospondin 2	0.68
*TNC*	3371	Tenascin C	0.71
*VEGFC*	7424	Vascular endothelial growth factor C	0.78

**Table 4:** Genes positively correlated with *ADGRF5* expression in the colon of colorectal cancer patients belonging to cell adhesion molecules signaling pathway—hsa04514.

Gene Symbol	Gene ID	Gene Description	Spearman Correlation
*CD34*	947	CD34 molecule	0.90
*CD86*	942	CD86 molecule	0.65
*CDH2*	1000	Cadherin 2	0.63
*CDH5*	1003	Cadherin 5	0.92
*ESAM*	90952	Endothelial cell adhesion molecule	0.81
*ITGA4*	3676	Integrin subunit α4	0.74
*ITGAV*	3685	Integrin subunit αV	0.63
*ITGB1*	3688	Integrin subunit β1	0.61
*JAM2*	58494	Junctional adhesion molecule 2	0.74
*JAM3*	83700	Junctional adhesion molecule 3	0.79
*NCAM2*	4685	Neural cell adhesion molecule 2	0.57
*NLGN4X*	57502	Neuroligin 4 X-linked	0.70
*PDCD1LG2*	80380	Programmed cell death 1 ligand 2	0.67
*PECAM1*	5175	Platelet and endothelial cell adhesion molecule 1	0.83
*PTPRC*	5788	Protein tyrosine phosphatase receptor type C	0.59
*PTPRM*	5797	Protein tyrosine phosphatase receptor type M	0.83
*SDC2*	6383	Syndecan 2	0.72
*SELE*	6401	Selectin E	0.70
*SELL*	6402	Selectin L	0.59
*VCAM1*	7412	Vascular cell adhesion molecule 1	0.74
*VCAN*	1462	Versican	0.77

**Table 5:** Genes negatively correlated with *ADGRF5* expression in the colon of colorectal cancer patients belonging to ribosome signaling pathway—hsa03010.

Gene Symbol	Gene ID	Gene Description	Spearman Correlation
*FAU*	2197	FAU ubiquitin-like and ribosomal protein S30 fusion	−0.36
*MRPL1*	65008	Mitochondrial ribosomal protein L1	−0.37
*MRPL2*	51069	Mitochondrial ribosomal protein L2	−0.30
*MRPL3*	11222	Mitochondrial ribosomal protein L3	−0.30
*MRPL4*	51073	Mitochondrial ribosomal protein L4	−0.37
*MRPL11*	65003	Mitochondrial ribosomal protein L11	−0.39
*MRPL12*	6182	Mitochondrial ribosomal protein L12	−0.44
*MRPL14*	64928	Mitochondrial ribosomal protein L14	−0.38
*MRPL15*	29088	Mitochondrial ribosomal protein L15	−0.29
*MRPL16*	54948	Mitochondrial ribosomal protein L16	−0.35
*MRPL17*	63875	Mitochondrial ribosomal protein L17	−0.37
*MRPL20*	55052	Mitochondrial ribosomal protein L20	−0.36
*MRPL21*	219927	Mitochondrial ribosomal protein L21	−0.38
*MRPL22*	29093	Mitochondrial ribosomal protein L22	−0.39
*MRPL23*	6150	Mitochondrial ribosomal protein L23	−0.38
*MRPL24*	29093	Mitochondrial ribosomal protein L24	−0.41
*MRPL27*	51264	Mitochondrial ribosomal protein L27	−0.47
*MRPL32*	64983	Mitochondrial ribosomal protein L32	−0.34
*MRPL34*	64981	Mitochondrial ribosomal protein L34	−0.36
*MRPL36*	64979	Mitochondrial ribosomal protein L36	−0.38
*MRPS2*	51116	Mitochondrial ribosomal protein S2	−0.37
*MRPS5*	64969	Mitochondrial ribosomal protein S5	−0.33
*MRPS9*	64965	Mitochondrial ribosomal protein S9	−0.35
*MRPS12*	6183	Mitochondrial ribosomal protein S12	−0.41
*MRPS15*	64960	Mitochondrial ribosomal protein S15	−0.37
*MRPS16*	51021	Mitochondrial ribosomal protein S16	−0.40
*MRPS17*	51373	Mitochondrial ribosomal protein S17	−0.36
*MRPS18A*	55168	Mitochondrial ribosomal protein S18A	−0.29
*RPL3*	6122	Ribosomal protein L3	−0.29
*RPL5*	6125	Ribosomal protein L5	−0.31
*RPL6*	6128	Ribosomal protein L6	−0.33
*RPL7A*	6130	Ribosomal protein L7a	−0.35
*RPL8*	6132	Ribosomal protein L8	−0.30
*RPL11*	6135	Ribosomal protein L11	−0.33
*RPL12*	6136	Ribosomal protein L12	−0.37
*RPL13*	6137	Ribosomal protein L13	−0.40
*RPL14*	9045	Ribosomal protein L14	−0.41
*RPL15*	6138	Ribosomal protein L15	−0.34
*RPL18*	6141	Ribosomal protein L18	−0.42
*RPL18A*	6142	Ribosomal protein L18a	−0.29
*RPL19*	6143	Ribosomal protein L19	−0.38
*RPL23*	9349	Ribosomal protein L23	−0.31
*RPL23A*	6147	Ribosomal protein L23a	−0.33
*RPL24*	6152	Ribosomal protein L24	−0.43
*RPL27*	6155	Ribosomal protein L27	−0.37
*RPL27A*	6157	Ribosomal protein L27A	−0.37
*RPL28*	6158	Ribosomal protein L28	−0.35
*RPL29*	6159	Ribosomal protein L29	−0.42
*RPL31*	6160	Ribosomal protein L31	−0.33
*RPL32*	6161	Ribosomal protein L32	−0.42
*RPL34*	6164	Ribosomal protein L34	−0.34
*RPL35*	11224	Ribosomal protein L35	−0.39
*RPL35A*	6165	Ribosomal protein L35A	−0.38
*RPL36*	25873	Ribosomal protein L36	−0.43
*RPL37*	6167	Ribosomal protein L37	−0.31
*RPL37A*	6173	Ribosomal protein L37a	−0.37
*RPL38*	6169	Ribosomal protein L38	−0.39
*RPL39*	6170	Ribosomal protein L39	−0.36
*RPLP0*	6175	Ribosomal protein lateral stalk subunit P0	−0.39
*RPLP1*	6176	Ribosomal protein lateral stalk subunit P1	−0.36
*RPLP2*	6181	Ribosomal protein lateral stalk subunit P2	−0.44
*RPS2*	6187	Ribosomal protein S2	−0.42
*RPS3*	6188	Ribosomal protein S3	−0.34
*RPS4X*	6191	Ribosomal protein S4X	−0.31
*RPS6*	6194	Ribosomal protein S6	−0.29
*RPS7*	6201	Ribosomal protein S7	−0.35
*RPS8*	6202	Ribosomal protein S8	−0.38
*RPS9*	6203	Ribosomal protein S9	−0.35
*RPS10*	6204	Ribosomal protein S10	−0.34
*RPS11*	6205	Ribosomal protein S11	−0.35
*RPS13*	6207	Ribosomal protein S13	−0.38
*RPS14*	6208	Ribosomal protein S14	−0.40
*RPS15*	6209	Ribosomal protein S15	−0.39
*RPS15A*	6210	Ribosomal protein S15A	−0.31
*RPS16*	6217	Ribosomal protein S16	−0.39
*RPS17*	6218	Ribosomal protein S17	−0.30
*RPS18*	6222	Ribosomal protein S18	−0.34
*RPS19*	6223	Ribosomal protein S19	−0.34
*RPS21*	6227	Ribosomal protein S21	−0.29
*RPS23*	6228	Ribosomal protein S23	−0.30
*RPS24*	6229	Ribosomal protein S24	−0.31
*RPS25*	6230	Ribosomal protein S25	−0.33
*RPS27A*	6233	Ribosomal protein S27A	−0.33
*RPS29*	6235	Ribosomal protein S29	−0.31
*RPSA*	3921	Ribosomal protein SA	−0.36

## Data Availability

Data is contained within the article.
